# Interaction of Osteoarthritis and BMI on *Leptin* Promoter Methylation in Taiwanese Adults

**DOI:** 10.3390/ijms21010123

**Published:** 2019-12-23

**Authors:** Tzi-Peng Yang, Hsiao-Mei Chen, Chao-Chin Hu, Li-Yuan Chen, Fen-Fen Shih, Disline Manli Tantoh, Kuan-Jung Lee, Yi-Chia Liaw, Rong-Tzong Tsai, Yung-Po Liaw

**Affiliations:** 1 Department of Medical Laboratory and Biotechnology, Chung Shan Medical University, Taichung 40201, Taiwan; zpyang@csmu.edu.tw; 2 Department of Nursing, Chung Shan Medical University, Taichung 40201, Taiwan; fionajunlom@gmail.com (H.-M.C.); ritajack1107@gmail.com (F.-F.S.); 3 Department of Medical Applied Chemistry, Chung Shan Medical University, Taichung 40201, Taiwan; hucc@csmu.edu.tw; 4 Department of Physical Therapy, Chung Shan Medical University, Taichung 40201, Taiwan; cato@csmu.edu.tw; 5 Department of Medical Imaging, Chung Shan Medical University Hospital, Taichung 40201, Taiwan; tantohdisline@yahoo.com; 6 Department of Public Health and Institute of Public Health, Chung Shan Medical University, Taichung 40201, Taiwan; jasminemachi@gmail.com; 7 Division of Neurology, Neurology Institute, Taipei Veterans General Hospital, Taipei 112, Taiwan; ycliaw1201@gmail.com; 8 Institute of Medicine, Chung Shan Medical University, Taichung 40201, Taiwan; 9 Institute of Biochemistry, Microbiology and Immunology, Chung Shan Medical University, Taichung 40201, Taiwan

**Keywords:** BMI, *LEP*, obesity, methylation, osteoarthritis

## Abstract

*Leptin* (*LEP*) regulates glucose metabolism and energy storage in the body. Osteoarthritis (OA) is associated with the upregulation of serum *LEP*. *LEP* promoter methylation is associated with obesity. So far, few studies have explored the association of BMI and OA with *LEP* methylation. We assessed the interaction between body mass index (BMI) and OA on *LEP* promoter methylation. Data of 1114 participants comprising 583 men and 558 women, aged 30–70 years were retrieved from the Taiwan Biobank Database (2008–2015). Osteoarthritis was self-reported and cases were those who reported having ever been clinically diagnosed with osteoarthritis. BMI was categorized into underweight, normal weight, overweight, and obesity. The mean *LEP* promoter methylation level in individuals with osteoarthritis was 0.5509 ± 0.00437 and 0.5375 ± 0.00101 in those without osteoarthritis. The interaction between osteoarthritis and BMI on *LEP* promoter methylation was significant (*p*-value = 0.0180). With normal BMI as the reference, the mean *LEP* promoter methylation level was significantly higher in obese osteoarthritic individuals (*β* = 0.03696, *p*-value = 0.0187). However, there was no significant association between BMI and *LEP* promoter methylation in individuals without osteoarthritis, regardless of BMI. In conclusion, only obesity was significantly associated with *LEP* promoter methylation (higher levels) specifically in osteoarthritic patients.

## 1. Introduction

*Leptin* is a peptide hormone composed of 167 amino acids. It is mainly produced in the adipose tissue but could also be found in the placenta, mammary gland, and other body tissues [1]. Its major role in glucose metabolism was demonstrated in 1995 on ob/ob (*leptin-*deficient) mice that were extremely obese and diabetic [2]. *LEP* is a marker for energy storage and body weight; circulating *LEP* levels are proportional to body fat content [3,4]. Despite the high levels of *LEP* and energy stores in obese individuals, sensitivity to *lep*tin is decreased, resulting in an inability to detect satiety [5]. Energy deficiencies are associated with decreased *LEP* levels and *LEP* receptor activation in the arcuate nucleus of the hypothalamus [6].

Osteoarthritis also called degenerative joint disease is the commonest form of arthritis and is the main cause of pain, disability, and poor quality of life among older adults [7,8]. In Taiwan, about 37 % of individuals over 50 years old have OA [7]. Obesity is a well-established risk factor for OA [9]. The risk of OA higher in type 2 diabetic patients and obese individuals with metabolic syndrome [8].

*LEP* is one of the main regulators of osteoarthritis pathogenesis [10,11,12,13]. In a study conducted in China, serum *LEP* levels were significantly higher in osteoarthritic patients with or without metabolic syndrome, suggesting the regulatory role of *LEP* in OA progression [10]. *LEP* levels were also higher in the cartilage of osteoarthritic individuals than in those without OA [10,14]. *LEP* enhances osteoarthritis development by exerting pro-inflammatory and pro-catabolic actions on the cartilage, leading to articular degeneration which is typical of osteoarthritis [15]. High levels of *LEP* expressions promote the synthesis and expression of nitric oxide, matrix metalloprotease-9, and matrix metalloprotease-13 in chondrocytes, thereby affecting their functions which could finally result in osteoarthritis [11,16]. Systemic inflammatory impacts of adipokines including *LEP*, resistin, and adiponectin (ADIPO) among others mediate the relationship between obesity and osteoarthritis [17,18].

Recently, several researchers have explored genetic and epigenetic processes including single nucleotide polymorphism (SNP), methylation, and expression of *LEP* in obesity and OA [11,19,20,21,22,23]. For instance, in Wistar National Institute of Nutrition (WNIN) obese mutant rats, *LEP* methylation levels, and transcription levels were positively correlated, suggesting a complex and dynamic underlying epigenetic mechanism for aberrant *LEP* expression in obesity [20]. *LEP* promoter methylation is associated with gene expression [24]. Moreover, it is tissue-specific and differs in different stages of development in humans [25,26]. For instance, *LEP* promoter methylation levels in human chondrocytes were inversely correlated with *LEP* expression, with advanced osteoarthritic chondrocytes being more highly expressed than the minimally osteoarthritic and normal chondrocytes [12]. Moreover, *LEP* levels were significantly higher in advanced osteoarthritic cartilage compared to minimally osteoarthritic cartilage [13].

So far, few studies have been conducted to determine the interaction between BMI and osteoarthritis on *LEP* methylation. This study was conducted to investigate the interaction of BMI with osteoarthritis on *LEP* promoter methylation.

## 2. Results

Table 1 shows the basic characteristics of the study participants stratified by OA status. There were 1141 participants comprising 48 osteoarthritic and 1093 non-osteoarthritic cases. The mean ± standard error (SE) *LEP* promoter methylation level in osteoarthritic cases was 0.5509 ± 0.00437 and was significantly higher (*p*-value = 0.0064) than that (0.5375 ± 0.00101) in the non-cases. In addition, the ages of the cases were significantly different from those of the non-cases (*p*-value < 0.0001). However, BMI, sex, waist-hip ratio, exercise, smoking, alcohol drinking, diabetes, and hypertension were not significantly different between the osteoarthritic cases and non-osteoarthritic cases (Table 1).

Results of logistic regression analysis revealed no significant association of OA and BMI with *LEP* promoter methylation (Table 2). However, age was significantly associated with higher levels of *LEP* gene promoter methylation. The regression coefficients (*β*); *p*-value were 0.00931; 0.0002, 0.01984; <0.001, and 0.02581; <0.0001, for the age groups 41–50, 51–60, and 61–70 years, respectively (Table 2).

Even though the association of OA and BMI with *LEP* promoter methylation was not significant, a significant interaction (*p*-value = 0.018) between OA and BMI on *LEP* promoter methylation was observed (Table 3). After stratification by OA status, BMI was significantly associated with *LEP* promoter methylation only in osteoarthritic individuals (Table 3); obesity was significantly associated with higher levels of *LEP* promoter methylation in osteoarthritic individuals (*β* = 0.03696; *p*-value = 0.0187). In addition, overweight was associated (borderline significance) with higher *LEP* promoter methylation; *β* = 0.02045 and *p*-value = 0.536 (Table 3).

## 3. Discussion

To the best of our knowledge, this is the first study to investigate the interaction between BMI and osteoarthritis on *LEP* promoter methylation. In osteoarthritic patients, the mean *LEP* promoter methylation level was significantly higher in obese individuals. However, in non-osteoarthritic individuals, BMI was not significantly associated with *LEP* promoter methylation.

*LEP* promoter methylation is associated with gene expression [24]. In WNIN obese mutant rats, *LEP* methylation levels and transcription were positively correlated, suggesting a complex and dynamic underlying epigenetic mechanism for aberrant *LEP* expression in obesity [20]. In humans, *LEP* methylation levels were inversely correlated with gene expression [12]. In this regard, we assume that serum *LEP* levels in obese osteoarthritic individuals (whose *LEP* promoter methylation levels were significantly higher) could be lower than the levels in normal-weight osteoarthritic individuals. Generally, *LEP* levels are positively correlated with adiposity and are usually elevated in obese individuals [4,27,28,29]. However, low *LEP* levels have also been found to be associated with weight gain. For example, in a longitudinal study conducted on obese Pima Indians, participants who gained weight had lower mean plasma *LEP* levels compared to those with a stable weight, suggesting the positive association of low *LEP* levels with weight gain and obesity [30]. In addition, results from reanalyzes of data from previous clinical trials showed that obese individuals with low levels of *LEP* lost more weight after treatment with *LEP* analog (metreleptin), compared with the placebo. As such, using low *LEP* levels as biomarkers, *LEP* analogs or other receptor agonists could be developed as obesity therapy in a subgroup of obese individuals [31]. In this regard, the obese osteoarthritic individuals in our study assumed to have low levels of *leptin* might have a better response (reduced weight) to *leptin* therapy, compared with those with higher levels of *leptin*. Hence, OA could be indirectly targeted in individuals with low *leptin* levels by treating obesity which is its major risk factor [9]. Since epigenetic processes are reversible, epigenetic therapy has also been suggested as a possible therapeutic potential for the treatment of osteoarthritis [12]. Hence, methylation-associated osteoarthritis could be epigenetically managed with DNA methylation inhibitors.

Most studies have demonstrated that serum *leptin* levels in people with osteoarthritis are high [10,15,32]. In our study, *LEP* promoter methylation levels were higher in obese individuals with osteoarthritis. Aside from DNA methylation, transcription factors and SNP are also believed to influence *LEP* gene expression [11,19,22]. In addition, factors like insulin and *leptin* receptors and resistance also influence the serum levels of *leptin* [1,33,34]. However, it was beyond the scope of our study to explore all the complex processes that could affect the expression and function of *LEP* in OA.

The current advancement in precision medical research has paved the way to explore aging and epigenetic modification of aging-related genes [35]. For instance, serum levels of *LEP* were found to be high in aging and obesity-related diseases like obstructive sleep apnea (OSA) and Alzheimer’s disease [36,37]. Moreover, lower levels of *LEP* promoter methylation were associated with anorexia nervosa (AN) [38]. These show the importance of *leptin* in the regulation of aging and aging-related diseases. Osteoarthritis and obesity are two prevalent aging health problems in the world [39]. As previously stated, the incidence of osteoarthritis in obese individuals is high [32]. Our findings suggest that *LEP* methylation might be involved in the pathogenesis of osteoarthritis. According to the United Nations classification, Taiwan is an aging society [40]. The percentage of adults over 65 years old in Taiwan is forecast to surpass 20% by 2025 [7].

Even though LEP methylation data are available in the Taiwan Biobank Database, data on serum levels of LEP are not available in the database. As such, we could not assess the serum levels of LEP in the participants. This is a limitation of our study.

## 4. Materials and Methods

### 4.1. Data Source

All the data used in this study were retrieved from the Taiwan Biobank database (2008–2015). Enrollment in the Taiwan biobank is restricted to Taiwanese who are 30 to 70 years old without a personal history of cancer [41].

### 4.2. Study Participants

A total of 1141 individuals (583 men and 558 women) were eligible for this study. Their demographic data including sex, age, body mass index (BMI), and waist-hip ratio (WHR); lifestyle data (regular exercise, smoking, and alcohol consumption); disease history (diabetes and hypertension), and genetic information (*LEP* methylation) were obtained from the Taiwan Biobank Database. Information on osteoarthritis was self-reported and participants were grouped into two: non-cases were those who reported no prior clinical diagnosis of osteoarthritis while cases were those who reported having ever been clinically diagnosed with osteoarthritis. BMI categories included underweight (BMI < 18.5 kg/m^2^), normal weight (18.5 ≤ BMI < 24 kg/m^2^), overweight (24 ≤ BMI < 27 kg/m^2^), obesity (BMI ≥ 27 kg/m^2^).

### 4.3. DNA Methylation Analysis

DNA methylation assessment was performed on sodium bisulfite-treated DNA from whole blood using the Infinium^®^ MethylationEPIC BeadChipEPIC array (Illumina Inc. San Diego, CA, USA). The following quality control measures were taken: (1) correction for dye-bias across batches by normalization, (2) removal of background signals, (3) elimination of outliers by the median absolute deviation method, and (4) elimination of probes with poor detection (*p*-value > 0.05) and those whose bead counts were < 3. The Chung Shan Medical University Institutional Review Board (CS2-17070) approved this study.

### 4.4. Statistical Analysis

The SAS 9.4 software (SAS Institute, Cary, NC, USA) was used for data management and analysis. T-test was used to compare the mean *LEP* promoter methylation levels in osteoarthritic and non-osteoarthritic participants and the results were presented as mean ± standard error (SE). Chi-square test was used to compare the categorical variables between the osteoarthritic and non-osteoarthritic cases and the results were presented as percentages. Multivariate linear regression models were used to determine the association of osteoarthritis and BMI with *LEP* promoter methylation and the interaction between osteoarthritis and BMI on *LEP* promoter methylation. Because whole blood was used to determine DNA methylation, the Reference-Free Adjustment for Cell-Type composition (ReFACTor) method was used to adjust for cell-type heterogeneity [42].

## 5. Conclusions

BMI was significantly associated with higher *LEP* promoter methylation levels in individuals with osteoarthritis. This association was particularly prominent in obese osteoarthritic individuals.

## Figures and Tables

**Table 1:** Basic characteristics of participants stratified by osteoarthritis status.

Variable	Without Osteoarthritis (*n* = 1093)	Osteoarthritis (*n* = 48)	*p*-Value
*LEP* promoter mean	0.5375 ± 0.00101	0.5509 ± 0.00437	0.0064
BMI (kg/m^2^)			0.5978
Normal	516(47.21)	24(50.00)	
Underweight	36(3.29)	0(0.00)	
Overweight	319(29.19)	13(27.08)	
Obese	222(20.31)	11(22.92)	
Sex			0.8767
Men	534(48.86)	24(50.00)	
Women	559(51.14)	24(50.00)	
Age			<0.0001
30–40	286(26.17)	2(4.17)	
41–50	281(25.71)	5(10.42)	
51–60	327(29.92)	14(29.17)	
61–70	199(18.21)	27(56.25)	
Waist-hip ratio			0.1070
Men < 0.9; women < 0.8	470(43.00)	15(31.25)	
men ≥ 0.9; women ≥ 0.8	623(57.00)	33(68.75)	
Exercise			0.0422
No	618(56.54)	20(41.67)	
Yes	475(43.46)	28(58.33)	
Smoking			0.1175
Never	815(74.57)	38(79.17)	
Quit	156(14.27)	9(18.75)	
Current	122(11.16)	1(2.08)	
Drinking			0.4099
Never	980(89.66)	45(93.75)	
Quit	37(3.39)	2(4.17)	
Current	76(6.95)	1(2.08)	
Diabetes			0.0816
No	993(90.85)	40(83.33)	
Yes	100(9.15)	8(16.67)	
Hypertension			0.2351
No	874(79.96)	35(72.92)	
Yes	219(20.04)	13(27.08)	

**Table 2:** Multiple linear regression showing the association of *LEP* promoter methylation with osteoarthritis and BMI.

Variable	*β*	*p*-Value
Osteoarthritis (reference: no)		
Yes	0.00107	0.8097
BMI (reference: normal)		
Underweight	−0.00363	0.4820
Overweight	−0.00128	0.5497
Obese	−0.00198	0.4349
Sex (reference: women)		
Men	−0.00041	0.8502
Age (reference: 30–40)		
41–50	0.00931	0.0002
51–60	0.01984	<0.0001
61–70	0.02581	<0.0001
Waist-hip ratio (reference: male < 0.9; female < 0.8)		
Men ≥ 0.9; women ≥ 0.8	−0.00161	0.4389
Exercise (reference: no)		
Yes	−0.00148	0.4347
Smoking (reference: never)		
Quit	0.00029	0.9148
Current	0.00159	0.6063
Drinking (reference: never)		
Quit	0.00402	0.4184
Current	−0.00556	0.1302
Diabetes (reference: no)		
Yes	0.00099	0.7544
Hypertension (reference: no)		
Yes	−0.00001	0.9973

**Table 3:** Multiple linear regression showing the association between *LEP* promoter methylation and BMI stratified by osteoarthritis status.

Variable	Without Osteoarthritis		Osteoarthritis	
	*β*	*p*-Value	*β*	*p*-Value
BMI (reference: normal)				
Underweight	−0.00365	0.4803	-	-
Overweight	−0.00241	0.2739	0.02045	0.0536
Obese	−0.00311	0.2306	0.03696	0.0187
Sex (reference: female)				
Male	0.00018	0.9341	−0.01712	0.1392
Age (reference: 30–40)				
41–50	0.00907	0.0004	0.01646	0.5695
51–60	0.01993	<.0001	0.01252	0.6110
61–70	0.02544	<.0001	0.03016	0.2206
Waist-hip ratio (reference: male < 0.9; female < 0.8)				
Men ≥ 0.9; women ≥ 0.8	−0.00115	0.5853	−0.01395	0.2546
Exercise (reference: no)				
Yes	−0.00127	0.5111	−0.00193	0.8479
Smoking (reference: never)				
Quit	0.00009	0.9737	0.00112	0.9352
Current	0.00199	0.5226	−0.04526	0.1564
Drinking (reference: never)				
Quit	0.00211	0.6807	0.04499	0.0456
Current	−0.00580	0.1182	−0.01202	0.6944
Diabetes (reference: no)				
Yes	0.00151	0.6462	0.00381	0.7518
Hypertension (reference: no)				
Yes	−0.00020	0.9340	0.01222	0.2126
Osteoarthritis * BMI interaction	*p*-value = 0.0180			

- signifies no available data.

## Abbreviations

LEP	Leptin
OA	Osteoarthritis
BMI	Body mass index
WHR	Waist–hip ratio
SE	Standard error
β	Beta coefficient
